# Creative ways to well-being: Reappraisal inventiveness in the context of anger-evoking situations

**DOI:** 10.3758/s13415-016-0465-9

**Published:** 2016-09-28

**Authors:** Andreas Fink, Elisabeth M. Weiss, Ursula Schwarzl, Hannelore Weber, Vera Loureiro de Assunção, Christian Rominger, Günter Schulter, Helmut K. Lackner, Ilona Papousek

**Affiliations:** 1 Institute of Psychology, University of Graz, Universitaetsplatz 2/III, A-8010 Graz, Austria; 2 Institute of Psychology, University of Graz, Graz, Austria; 3 Institute of Psychology, University of Greifswald, Greifswald, Germany; 4 Institute of Physiology, Medical University of Graz, Graz, Austria

**Keywords:** Cognitive reappraisal, Creativity, Emotion, Anger, Alpha

## Abstract

Neuroscientific studies in the field of creativity mainly focused on tasks drawing on basic verbal divergent thinking demands. This study took a step further by investigating brain mechanisms in response to other types of creative behavior, involving more “real-life” creativity demands in the context of emotion regulation and well-being. Specifically, functional patterns of EEG alpha activity were investigated while participants were required to generate as many and as different ways as possible to reappraise presented anger-eliciting situations in a manner that reduces their anger. Cognitive reappraisal involves some of the same cognitive processes as in conventional verbal creativity tasks, inasmuch as it requires an individual to inhibit or disengage from an emotional event, to shift attention between different perspectives, and to flexibly adopt new solutions. To examine whether alpha oscillations during cognitive reappraisal are different from those during conventional creative ideation, the EEG was also assessed during performance of the Alternative Uses task, requiring individuals to generate as many and as original uses of an object as possible. While cognitive reappraisal was associated with a similar pattern of alpha power as observed in conventional verbal creative ideation, the former yielded significantly stronger alpha power increases at prefrontal sites, along with lower alpha increases at more posterior cortical sites, indicating higher cognitive control and less spontaneous imaginative thought processes in the generation of effective strategies to regulate an ongoing negative emotional state.

## Introduction

There is an increasing interest in the neuroscientific study of creativity. The emergence of ever more sophisticated psychometric research approaches, along with advanced neuroimaging techniques such as functional magnetic resonance imaging (fMRI) or the analysis of event-related (de)synchronization of brain activity in the electroencephalogram (EEG), have yielded valuable insights into potential brain correlates underlying various creativity-related demands (e.g., Fink & Benedek, 2014; Gonen-Yaacovi et al., 2013). Most of the studies in this emerging field focused on divergent thinking ability, which is considered a reliable and useful indicator of creative potential (Runco & Acar, 2012). In the present study, we extended the focus of creativity research toward the generation of cognitive reappraisals, which implies the operation of creativity-related processes in an affective context. Cognitive reappraisal refers to deliberately viewing an emotionally evocative event from a different perspective and re-interpreting its meaning, thereby changing its emotional impact (Lazarus & Alfert, 1964; Lazarus & Folkman, 1984). It requires an individual to shift attention between different perspectives and to flexibly adopt new situations or strategies, and relies on basic executive functions such as the inhibition of highly activated or prepotent representations, memory updating, and cognitive switching (Joorman & Gotlib, 2010; Malooly et al., 2013; Weber et al., 2014), which also play a major role in the generation of novel (creative) ideas to given open problems in non-emotional contexts (Beaty & Silvia, 2012; Benedek et al., 2012; Fink & Benedek, 2014; Gilhooly et al., 2007; Runco, 2010). While the overlap of cognitive processes between the generation of reappraisals and creative or divergent thinking is obvious and empirically substantiated (Weber et al., 2014), the generation of cognitive reappraisal, which per definition refers to the processing of emotionally relevant information, may require additional demands compared to divergent thinking in non-emotional contexts. These may also be reflected at the level of the brain.

Cognitive reappraisal is regarded as a particularly effective strategy in coping with adverse events (e.g., Augustine & Hemenover, 2009; Webb et al., 2012), and is thought to have positive implications for psychological health and well-being (e.g., Garnefski et al., 2002; Gross & John 2003). Consequently, encouraging patients to use cognitive reappraisal constitutes the core of modern psychotherapeutic approaches. The identification of neural correlates of and associated core functions participating in the generation of reappraisals may help to develop effective, evidence-based training strategies. Moreover, when the capacity for cognitive reappraisal is impaired, for instance in older people as a result of decline in relevant brain functions, training of other emotion regulation strategies may be more effective (see e.g., Smoski et al., 2014).

Gonen-Yaacovi et al. (2013) recently conducted a quantitative meta-analysis of functional imaging studies involving various creativity-related task demands. They identified a widespread neural network primarily involving frontal and parieto-temporal brain regions that may support cognitive processes implicated in diverse creativity-related task demands. Specifically, this “core creativity network” included regions of the lateral prefrontal cortex, which has consistently been found to be implicated in various higher order executive processes such as fluency, flexibility, or cognitive control (Gonen-Yaacovi et al., 2013). In addition, it included a set of brain regions (i.e., left angular, superior temporal, and inferior frontal gyri) which have been associated with semantic processes such as the activation and retrieval of internal memory representations (Binder et al., 2009). Similarly, functional imaging studies investigating brain activation patterns after participants were instructed to use reappraisal to reduce the negative impact of emotional pictures consistently showed increased activation in the lateral prefrontal cortex, particularly during earlier periods of the experimental reappraisal phases that were presumably dominated by efforts to generate alternative appraisals (compared to later periods that were more dominated by maintenance processes; Dillon & Pizzagalli, 2013; Kalisch, 2009, Ochsner et al., 2002; Phan et al., 2005). At the same time, activation in multiple other cortical regions was typically observed, but obviously not all of these activations were linked to processes specifically implicated in the generation of cognitive reappraisals (Phan et al., 2005). Using more specific experimental paradigms comparing reappraisal to other coping strategies such as expressive suppression and distraction, recent studies supported the particular involvement of (especially left) ventral and rostral regions of the prefrontal cortex in the generation of cognitive reappraisals, in line with the localization of relevant executive functions (Dörfel et al., 2014; Price et al. 2013).

Fink and Benedek (2014) reviewed EEG studies on creativity-related demands, specifically looking at studies in which similar tasks (i.e., tasks involving verbal creative ideation) and neurophysiological indices of brain activity (i.e., alpha power measures) were used. Their review revealed a largely consistent pattern of results, highlighting the important role of EEG alpha band activity in verbal divergent thinking demands. Specifically, alpha activity has been found to vary as a function of creativity-related task demands (the more the task draws on creativity-related demands the higher the level of alpha power) and as a function of an individuals’ creativity level (more creative individuals show more alpha power). In addition, alpha power has also been found to be sensitive to verbal creativity interventions (Fink et al., 2006, 2011) and, most intriguingly, Lustenberger et al. (2015) even demonstrated increases in psychometrically determined creativity (assessed via the well-known Torrance Tests of Creative Thinking; Torrance, 1966) as a result of 10-Hz transcranial alternating current stimulation. Creativity-related effects in the alpha frequency band have been observed in the entire alpha frequency range (~8–12 Hz), both in the lower (~ 8–10 Hz) and in the upper (~10–12 Hz) alpha sub-bands, though somewhat more pronounced in the upper alpha band (Fink & Benedek, 2014). Increases in alpha power have been interpreted to reflect the absence of stimulus-driven, external bottom-up stimulation. Thus, alpha power indicates a state of high internal, task-focused processing demands (Benedek et al., 2011, 2014; Klimesch et al., 2007; von Stein & Sarnthein, 2000), facilitating cognitive control and effective search and retrieval of internal memory representations (Fink & Benedek, 2014).

Taken together, existing EEG and fMRI studies revealed that creative ideation is reliably associated with activity in a widespread neural network primarily involving frontal and posterior parietal brain regions, which are known to be important components of a neural network specialized for attention, working memory, and semantic information processing (Fink & Benedek, 2014; Gonen-Yaacovi et al., 2013). Meanwhile, there is large consensus that creative cognition is organized in widespread neural networks, including both regions associated with executive processes and cognitive control, and regions closely linked with (at least a subset of) the default network of the brain (e.g., Beaty et al., 2015, 2016; Jung et al., 2013). Such findings provide strong evidence for the idea that creativity is the result of efficient cooperation between cognitive control processes and spontaneous thought (Beaty et al., 2015; Mok, 2014; for an integrated framework on brain networks and creative cognition see Beaty et al., 2016).

While our understanding of potential creativity-related brain processes continuously increases, one might criticize the fact that most of the neuroscientific studies in this field used tasks involving verbal divergent thinking (such as the generation of alternative uses of everyday objects). Some promising attempts to investigate brain mechanisms during the performance of other types of creative behavior also involving more “real-life” creativity demands have been reported by, e.g., Aziz-Zadeh et al. (2013) or Kenett et al. (2015), who investigated visual divergent-thinking demands. Bengtsson et al. (2007), Berkowitz and Ansari (2010), Ellamil et al. (2012), and Kowatari et al. (2009) focused on the artistic creativity domain, studying brain activity during musical improvisation, visual art, and designing book covers or new pens, respectively (for further details see Gonen-Yaacovi et al., 2013; for related behavioral papers see also Beaty et al., 2014 and Okuda et al., 1991). Beaty (2015) recently reviewed fMRI studies on musical improvisation and, quite similar to the existing divergent thinking literature, characterized the complex process of musical improvisation as being supported by large-scale brain networks responsible for both spontaneous imaginative thought processes and cognitive control.

Taking an additional, novel step forward, the present study investigated functional patterns of EEG brain activity while participants were required to be creative in an affective context. On the one hand, this was motivated by the goal to study brain mechanisms in response to other types of creative behavior, including more “real-life” creativity demands, especially in the context of emotion regulation and well-being; and, on the other hand, this research was stimulated by our general intention to combine research from the affective and cognitive research traditions, which are often treated as separate rather than intertwining domains.

While functional imaging studies suggested the particular involvement of lateral parts of the prefrontal cortex in cognitive reappraisal, the investigation of task-related alpha power changes during the generation of cognitive reappraisals, which have extensively been investigated and have yielded a highly consistent picture in the field of creativity, is entirely novel. Specifically, in the present study changes in alpha power were analyzed while individuals were instructed to generate as many and as different appraisals of self-relevant negative emotional events. To this end, a modified version of the Reappraisal Inventiveness Test (RIT; Weber et al., 2014) was employed, in which participants are instructed to empathize with anger-eliciting situations and to think of as many different ways as possible to reappraise these in a manner that reduces their anger. The generation of reappraisals appears to be closely linked to concepts from the realms of creativity and divergent thinking (Joormann & Gotlib, 2010; Malooly et al., 2013; Weber et al., 2014). In the *plant* item of the RIT, for instance, participants are confronted with the following situation: “*You arrive at your apartment after having been on a long vacation. You had asked a friend of yours to water your plants while you were gone. Now you see that most of your plants have died. You call your friend. She tells you on the phone that the distance to your apartment was too far for her to water your plants as agreed*.” (Weber et al., 2014, p. 360). A person may reappraise this situation in a manner that she/he is now happy to have more room in her or his apartment, she/he may be relieved that she/he did not entrust the friend her/his dog for care, or she/he may take efforts to rescue the plants or even claim compensation from the friend. Hence, this task requires an individual to flexibly adopt and to generate new perspectives, solutions, or strategies, accompanied by overcoming and inhibiting the typical and most obvious response elicited by this situation (i.e., experience of anger). Such flexible idea production is likewise seen in many creativity-related task demands and, in fact, performance in the RIT has been found to be significantly associated with conventional divergent thinking measures and with the personality dimension of openness that is closely linked to creativity (Weber et al., 2014). Specifically, Weber et al. (2014) found that RIT performance was significantly associated with verbal divergent-thinking ability as assessed by means of the verbal imagination subscales of the German Berlin Intelligence Structure (BIS) test, a widely used measure of verbal divergent thinking ability (correlations up to *r* = .61, see Weber et al., 2014).

To examine whether or to what extent EEG alpha power changes during the generation of reappraisals are different from those during conventional creative idea generation, the EEG was also assessed during performance of the classic alternative uses task (AUT), requiring individuals to generate as many and as original uses of given everyday objects. This task has been associated with strong alpha power increases at prefrontal sites, and a diffuse pattern of alpha power increases at more posterior (parietal) cortical sites. This pattern of finding has been interpreted as reflecting a state of heightened internally focused attention (Benedek et al., 2011, 2014; Klimesch et al., 2007; von Stein & Sarnthein, 2000), facilitating effective research and retrieval of relevant information from internal memory representations (Fink & Benedek, 2014). Since both the AUT and the RIT share various cognitive processes such as the inhibition of prevalent/typical responses or the effective search and retrieval of relevant information from memory, we might generally expect a similar pattern of alpha activity in both tasks. However, given that the RIT requires novel idea generation in an affective context, we might also expect observing some brain activity patterns that are specific to this task. Cognitive reappraisal of emotion-eliciting situations requires participants to inhibit (disengage from) negative emotional aspects of a situation, to switch (shift focus) between negative emotional and neutral mental sets when processing and re-interpreting the meaning of the situation, and to alter and update its affective value and motivational relevance (Malooly et al., 2013). Thus, through drawing on the participating executive functions in an affective context, some additional demands may be involved in the generation of cognitive reappraisals as compared to divergent thinking without emotional component. The associated cognitive control processes are closely linked to functions of the prefrontal cortex (Kalisch, 2009; Ochsner & Gross, 2005, 2008). Therefore, we expected prefrontal alpha activity, which is also thought to indicate top-down control by inhibiting task-irrelevant activity such as irrelevant sensory processing or the retrieval of interfering information (Klimesch et al., 2007; Sauseng et al., 2005), to be additionally increased during the generation of cognitive reappraisals compared to divergent thinking without emotional component.

## Method

### Participants

The sample comprised 81 healthy, right-handed female university students in the age range between 18 and 35 years (*M* = 22.65, *SD* = 3.60). They were recruited via announcements at the university, mailing lists, and social networks. Participants received either €10 or course credit for their participation. To confirm right-handedness, a standardized hand-skill test (Hand Dominance Test; Papousek & Schulter, 1999; Steingrüber & Lienert, 1971) was used. A female-only sample was chosen in order to avoid any confounding effects produced by potential gender differences in emotion-related abilities or typical behavior (e.g., Domes et al., 2010; Freudenthaler & Papousek, 2013). Additionally, women may be more motivated to down-regulate anger than men for social reasons (Evers et al., 2005). The study was performed in accordance with the American Psychological Association's Ethics Code and the 1964 Declaration of Helsinki, and was approved by the local ethics committee. Participants gave their written consent to participate in the study.

### Experimental tasks

#### Generation of reappraisals

**Fig. 1:**
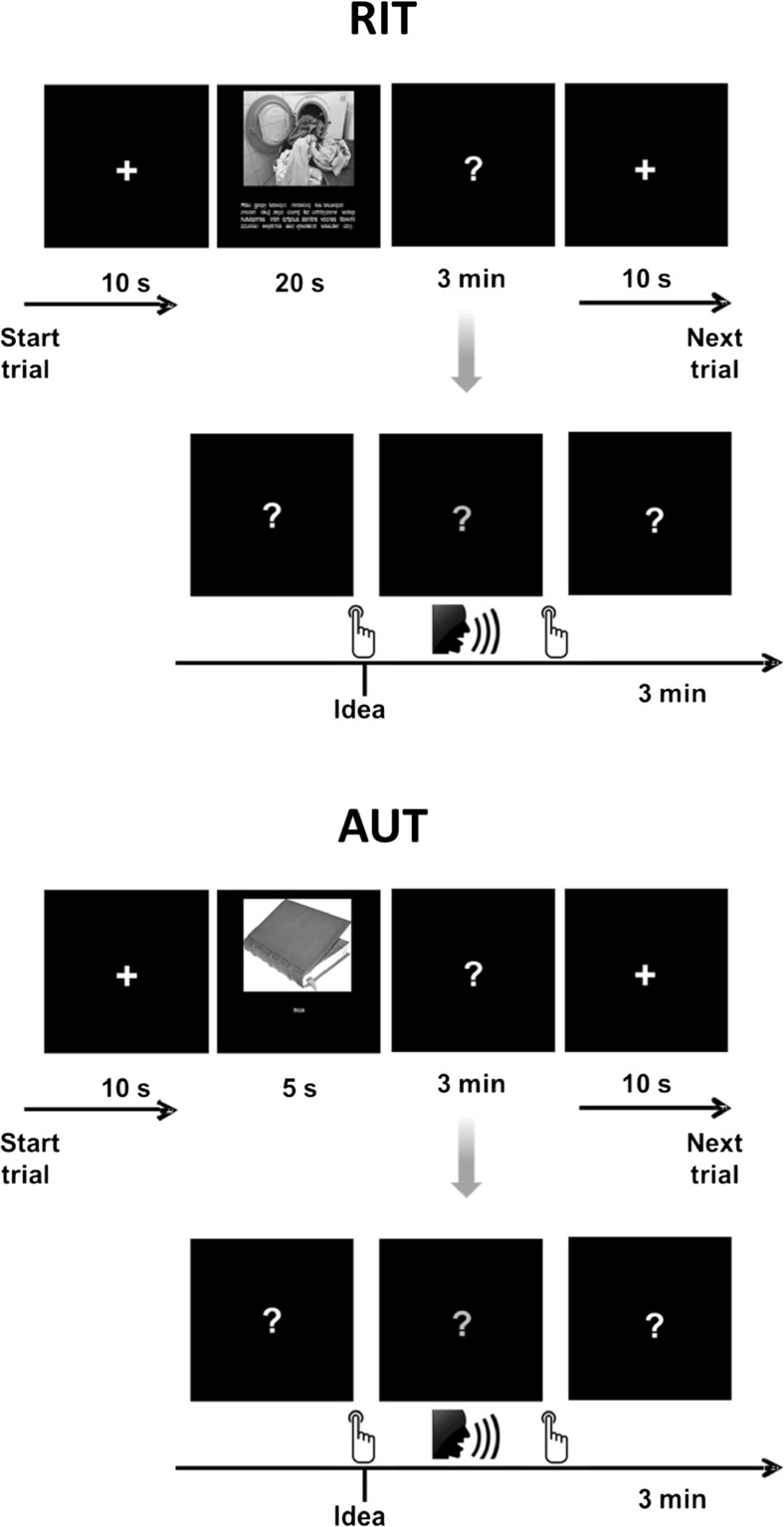
Overview of experimental tasks during EEG assessment. **Top**: Reappraisal Inventiveness Test (RIT); **bottom**: Alternative Uses Test (AUT)

The original version of the RIT (Weber et al., 2014) consists of four anger-eliciting vignettes that, in line with cognitive emotion theories, depict the behavior of another person who willingly or carelessly induces harm. Each vignette is supplemented by a matching photograph to make the situation more vivid. Participants are instructed to imagine the situation happening to them and to generate and write down as many different ways as possible to think about or appraise the situation in a way that diminishes anger. In this study, the items of the RIT were slightly adapted to incorporate them into the EEG environment: As shown in Fig. 1, each vignette was presented on a computer screen for 20 s. Participants were instructed to imagine the situation happening to them and to generate as many different ways as possible to think about or appraise the situation in a way that diminishes anger. They were instructed to press a button whenever a new appraisal came to mind, and to vocalize the idea concisely in one or two short sentences immediately after pressing the button. They were then asked to press the button again, and the task was resumed until the allotted time of 3 min had elapsed (note that the idea-generation window including all response phases was restricted to 3 min). In doing so, we were able to separate EEG segments related to the production of reappraisals from segments contaminated with the production of speech. This protocol has proved to be particularly suitable in previous research in the creativity domain (Fink et al., 2007). The allotted time of 3 min for each item corresponded to the original procedure of the RIT. Participants’ vocal responses were audiotaped for later analysis.

The protocol comprised eight vignettes, the four original vignettes of the RIT (Weber et al., 2014), and four additional vignettes that were constructed and pre-tested in pilot tests in order to match the main characteristics of the original vignettes to the best possible extent. Pilot tests revealed that the newly constructed vignettes were highly comparable to the original test items, both with respect to the number of generated reappraisals and the number of categorically different responses. As outlined in the test manual (Weber et al., 2014), RIT-fluency refers to the total number of generated non-identical ideas that qualified as cognitive reappraisals. RIT-flexibility refers to the number of categorically different reappraisals (for the category scheme see Weber et al., 2014). In this study, all responses were independently rated by two experimenters, as suggested by the test manual. Inter-rater reliabilities (intra-class correlation (ICC)) for RIT-fluency (number of non-identical reappraisals) were *ICC* = .93, and for RIT flexibility (number of categorically different reappraisals) *ICC* = .93. After completing all RIT items participants were instructed to retrospectively rate the extent of anger they would experience when confronted with the situations depicted in the vignettes on a 7-point scale ranging from 0 (not angry at all) to 6 (extremely angry).

#### Alternative Uses Test (AUT)

The AUT, which is among the most commonly applied tasks in the neuroscientific study of creativity (Fink & Benedek, 2014), requires participants to think of as many different original uses for conventional objects (such as a brick or a barrel) as possible. Eight items were selected from previous studies (e.g., Fink et al., 2012) in order to match them to the reappraisal items. In addition, stimulus words in the AUT were presented conjointly with images of the respective objects, in order to make the task as comparable as possible to the RIT. Participants were instructed to generate as many and as original uses for the objects as possible, and to press the IDEA button whenever they became aware of an idea. Analogous to the RIT, they were asked to vocalize the idea immediately after pressing the button, after which the task was resumed until the allotted time of 3 min had elapsed (see Fig. 1). The AUT provides scores for fluency (number of generated non-redundant ideas) and originality. Originality was assessed by five raters who – after a thorough and comprehensive instruction – evaluated each single idea of the participants on a five-point rating scale ranging from 1 (“highly original”) to 5 (“not original at all”). The obtained ratings were averaged over all responses of a participant, so that one originality measure was available for each participant. Inter-rater agreement was satisfactory (*ICC*: .73). For greater clarity, the scale of the originality ratings was inverted for all further analyses, with higher scores (maximum of 5) now indicating higher originality.

### EEG recording and quantification

**Fig. 2:**
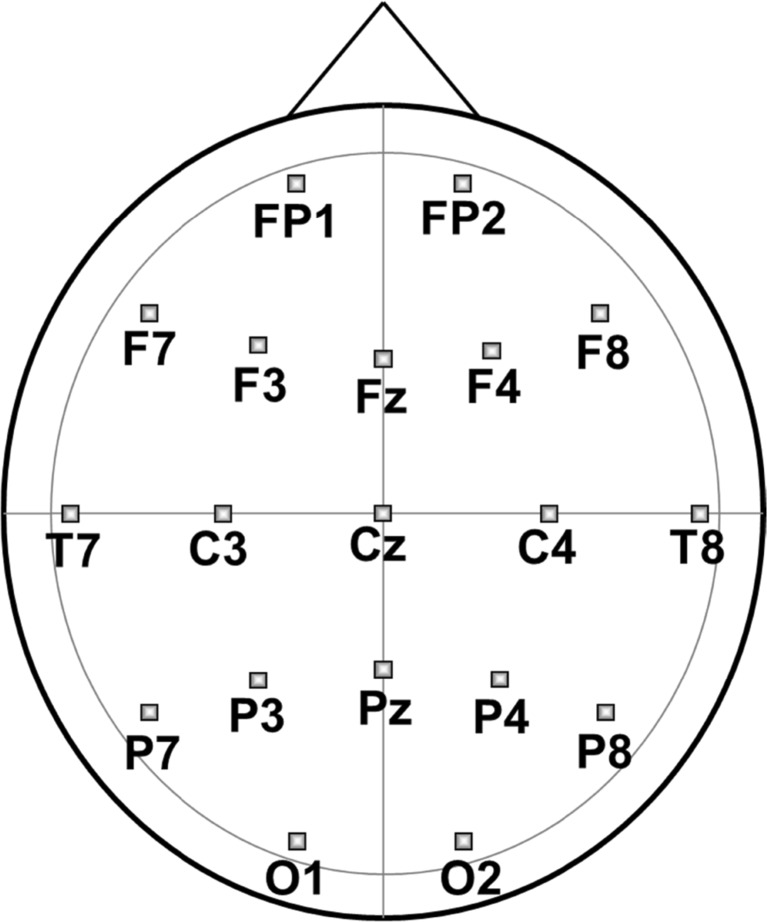
Placement of electrodes. *FP* frontopolar, *F* frontal, *T* temporal, *C* central, *P* parietal, *O* occipital

The EEG was measured with a Brainvision BrainAmp Research Amplifier (Brain Products) by means of Ag/AgCl electrodes located in an electrode cap in 19 positions (FP_1_, FP_2_, F_3_, F_7_, F_Z_, F_4_, F_8_, C_3_, C_Z_, C_4_, T_7_, T_8_, P_3_, P_Z_, P_4_, P_7_, P_8_, O_1_, O_2_; see Fig. 2); the ground electrode was located at FP_Z_, the reference electrode was placed on the nose. To register eye movements, an electro-oculogram (EOG) was recorded bipolarly between two electrodes diagonally placed above and below the inner and outer canthus of the right eye. The EEG signals were filtered between 0.1 Hz and 100 Hz; an additional 50-Hz notch filter was applied. Electrode impedances were kept below 5 kΩ for the EEG and below 10 kΩ for the EOG. All signals were sampled at a frequency of 500 Hz.

EEG data were preprocessed by removing drifts and low-pass filtering (50 Hz). The data were visually inspected for artifacts and artifactual epochs caused by muscle tension, eye blinks, or eye movements were excluded from further analyses. Similar to previous studies (e.g., Benedek et al., 2014; Fink et al., 2007, Fink, et al. 2009a, b, 2011; Jaarsveld et al., 2015; Schwab et al., 2014), we quantified task-related power (TRP) changes in the upper alpha band (10–12 Hz) during performance of the experimental tasks relative to the pre-stimulus reference period. The term “task-related” refers to the fact that brain activity during the performance of a given cognitive task (here: generation of creative ideas) is related to a pre-stimulus reference interval during which no task is performed (Pfurtscheller & Aranibar, 1977). This ensures that any differences in event- or task-related brain activity are due to the effect of group or task and not to differences in baseline brain activity. Task- or event-related changes in alpha power have been investigated in a variety of studies covering a broad range of different cognitive task demands (for overview see, e.g., Neuper & Klimesch, 2006), and they were found to be especially sensitive to cognitive task performance and higher cognitive abilities (e.g., Neubauer et al., 2006; Neubauer & Fink, 2009).

In this study, 8-s time segments (out of 10 s) of the reference period (starting 1,000 ms after the onset of the fixation cross), and 1,000-ms time windows directly before pressing the IDEA-button (1,250–250 ms) during performance of the AUT and the RIT were used as reference and activation intervals, respectively. Power estimates were obtained by squaring band pass filtered EEG signals, and then band power values (μV^2^) were averaged (horizontally, i.e. across time) for each time segment in the reference and the activation intervals. Then the TRP was calculated for each generated idea in the RIT and in the AUT and for each electrode position (i) by subtracting the log-transformed power during the reference interval from the log-transformed power during the activation interval according to the formula: TRP (logPow_i_) = log [Pow_i activation_] − log [Pow_i reference_] (Pfurtscheller, 1999). Finally, the TRP values were averaged across the total of generated ideas of the RIT and the AUT, respectively. Task-related increases in power from the reference to the activation period are reflected in positive values, whereas negative TRP values indicate decreases in power.

### Procedure

After completing the handedness test, participants were seated in an acoustically and electrically shielded examination room, and electrodes were attached. After a 2-min rest period, participants completed the AUT and the RIT, in a counterbalanced order. Following the RIT, participants completed the retrospective anger ratings for each of the depicted situations using the computer mouse. After the EEG recording, participants completed various psychometric tests for purposes related to other, non-overlapping research questions.

### Statistical analyses

To investigate potential TRP differences between the experimental tasks, a GLM for repeated measures involving the factors TASK (AUT vs. RIT), HEMISPHERE (left vs. right), and AREA (eight electrode positions in each hemisphere) was performed on the TRP in the upper alpha band. To test whether any task differences were already present at baseline (i.e., pre-stimulus reference interval), a GLM (TASK, HEMISPHERE, and AREA) was performed on the alpha power measures during the reference interval. To assess whether or to which extent alpha power changes during the RIT and the AUT were modulated by individual differences in task performance, separate GLMs for the RIT and the AUT were computed in considering AREA and HEMISPHERE as within-subjects factors and the AUT performance measures (fluency, originality) or the RIT performance scores (fluency, flexibility) as continuously distributed between-subjects factors (a separate GLM was conducted for each of the performance variables). In addition, a GLM for repeated measures with HEMISPHERE and AREA as within-subjects factors and the subjectively experienced ANGER during performing the RIT as continuously distributed between-subjects factor was computed for the task-related alpha power changes during the RIT. Finally, intercorrelations among the performance measures are reported (Pearson correlations). To illustrate interaction effects between task-related alpha power changes and one of the continuously distributed between-subjects factors (task performance or anger), predicted TRP values were calculated for one standard deviation below and one standard deviation above the sample mean of task performance/anger scores using standard regression analysis. In case of violations of sphericity assumptions, the multivariate approach to repeated measurements variables was used (Vasey & Thayer, 1987). Post-hoc comparisons were performed using Tukey’s Honestly Significant Differences (HSD) test. Estimates of effect sizes are given in terms of partial eta-squared measures (η_p_^2^). All statistical tests were performed with α = .05 (two-tailed).

## Results

### Task-related changes in EEG alpha power during performance of the RIT and the AUT: Overall comparison of tasks

**Fig. 3:**
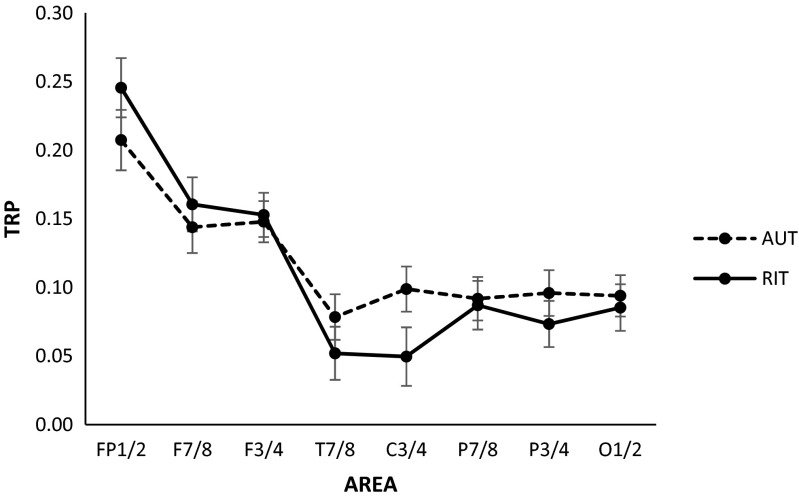
Task-related changes in alpha power (TRP) during peformance of the Reappraisal Inventiveness Test (RIT) and the Alternative Uses Test (AUT). Positive TRP values indicate increases in alpha power from the reference to the activation period

The three-way GLM analysis yielded a significant main effect of AREA (*F* (7, 74) = 14.75, *p* < .01, *η*_*p*_^*2*^ = .29), suggesting that the generation of alternative uses of objects (AUT) and reappraisals (RIT) was associated with comparatively strong alpha power increases at frontal, particularly frontopolar sites (as verified by Tukey post hoc tests; see Fig. 3). In addition, the main effect HEMISPHERE (*F* (1, 80) = 28.80, *p* < .01, *η*_*p*_^*2*^ = .27), along with the interaction between AREA and HEMISPHERE, were significant (*F* (7, 74) = 5.86, *p* < .01, *η*_*p*_^*2*^ = .12), indicating that at more posterior cortical sites alpha power increases were more pronounced in the right than in the left hemisphere. Tukey HSD test yielded significant hemispheric differences at all sites (F7 vs. F8: 0.13 vs. 0.17; T7 vs. T8: 0.03 vs. 0.10; C3 vs. C4: 0.05 vs. 0.10; P7 vs. P8: 0.06 vs. 0.12; P3 vs. P4: 0.06 vs. 0.11: O1 vs. O2: 0.07 vs. 0.11), except for FP_1_ versus FP_2_ (0.23 vs. 0.23) and F_3_ versus F_4_ (0.15 vs. 0.15).

Most interestingly, as revealed by a significant TASK by AREA interaction (*F* (7, 74) = 3.15, *p* < .01, *η*_*p*_^*2*^ = .07), the AUT and the RIT were accompanied by different patterns of task-related alpha power increases. As shown in Fig. 3, the RIT elicited stronger alpha increases at frontopolar sites than the AUT (*p* < .05, as assessed by Tukey’s HSD), while the AUT was associated with stronger increases at central sites (*p* <.05). The three-way interaction between TASK, HEMISPHERE, and AREA was not significant (*F* (7, 74) = 0.58, *p* = .77, *η*_*p*_^*2*^ = .01).

The analysis of alpha power measures during the reference interval revealed no significant effects involving the factor TASK, suggesting that the observed TRP differences between the RIT and the AUT were due to task performance and not due to differences in baseline alpha power.

### Task-related changes in EEG alpha power during performance of the RIT and the AUT as a function of task performance

**Fig. 4:**
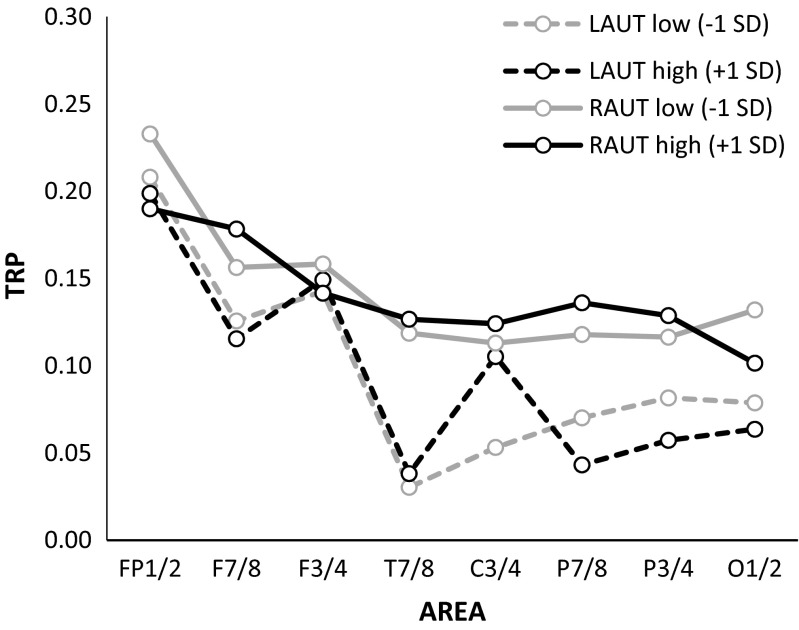
Task-related changes in alpha power (TRP) during performance of the Alternative Uses Test (AUT) as a function of task performance (AUT fluency). Positive TRP values indicate increases in alpha power from the reference to the activation period. Predicted TRPs (calculated using standard regression analysis) are plotted separately for each cortical position of the left (L) and the right (R) hemisphere for AUT performance changes one SD below (AUT low, dark gray lines) and one SD above (AUT high, black lines) the mean

Task performance had a significant effect on TRP only in the AUT, as reflected in a significant interaction between AREA, HEMISPHERE, and AUT fluency (*F* (7, 73) = 2.72, *p* < .05, *η*_*p*_^*2*^ = .03). Similar to previous EEG research on creative ideation (e.g., Benedek et al., 2014; Fink, et al. 2009a, b; Schwab et al., 2014), individuals scoring high on the AUT exhibited a more distinct pattern of hemispheric asymmetry with respect to task-related alpha power increases than lower scoring individuals, characterized by more pronounced increases over the right (vs. left) parietal cortex (see Fig. 4). The GLMs for the RIT yielded no significant effects involving the RIT performance scores (fluency, flexibility).

Supplementary analyses for the upper alpha power measures at baseline (pre-stimulus reference interval) revealed no significant effects involving the AUT fluency score.

### Task-related changes in alpha power during the RIT as a function of subjectively experienced anger

**Fig. 5:**
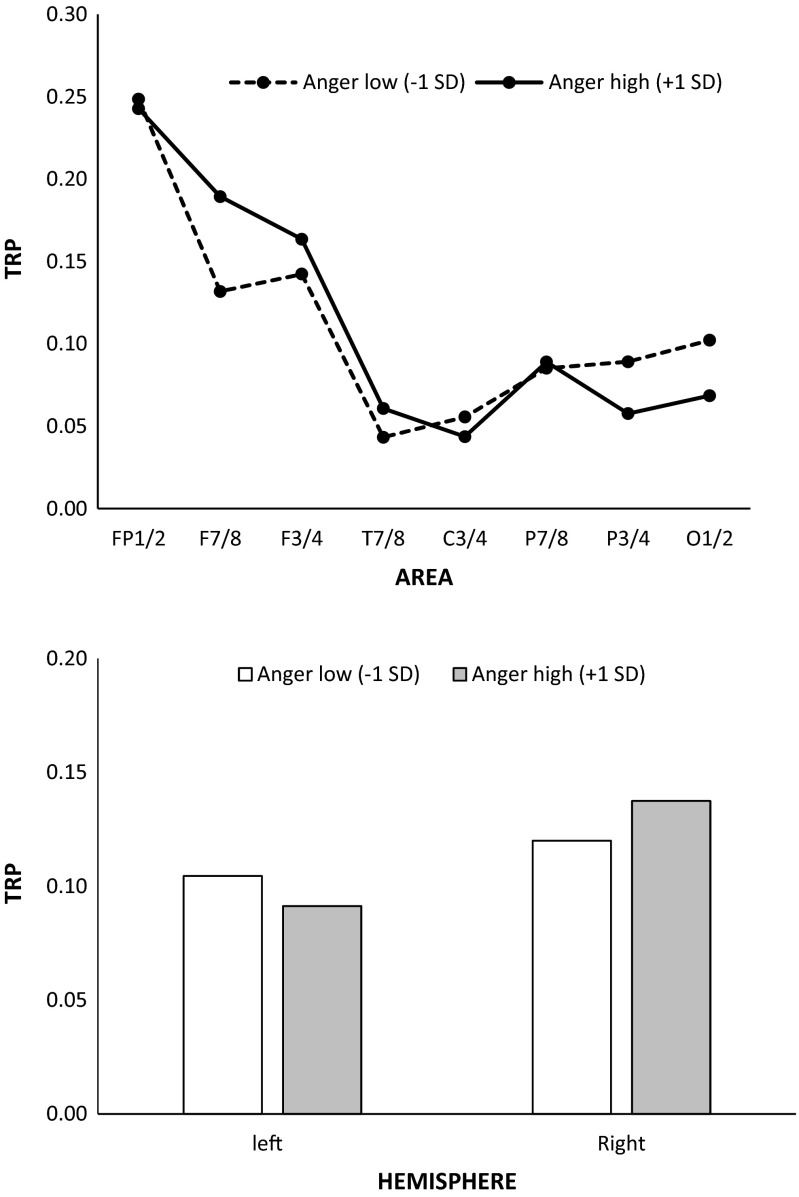
Task-related changes in alpha power (TRP) during performance of the Reappraisal Inventiveness Test (RIT) as a function of subjectively experienced anger. Positive TRP values indicate increases in alpha power from the reference to the activation period. **Top**: Interaction between ANGER and AREA; predicted TRPs (calculated using standard regression analysis) are plotted separately for each cortical position for changes of the anger ratings one SD below (Anger low) and one SD above (Anger high) the mean. **Bottom**: Interaction between ANGER and HEMISPHERE; predicted TRPs (calculated using standard regression analysis) are plotted separately for the left and the right hemisphere for changes of the anger ratings one SD below (Anger low) and one SD above (Anger high) the mean

Along with a significant main effect of AREA (*F* (7, 73) = 2.31, *p* < .05, *η*_*p*_^*2*^ = .03), the GLM revealed significant interactions between AREA and ANGER (*F* (7, 73) = 2.56, *p* < .05, *η*_*p*_^*2*^ = .02), and between HEMISPHERE and ANGER (*F* (1, 79) = 4.03, *p* < .05, *η*_*p*_^*2*^ = .05). As shown in Fig. 5, those individuals who experienced the presented RIT stimuli as being more anger evoking, exhibited stronger increases in alpha power at ventrolateral prefrontal sites (F_7/8_), and generally over the right hemisphere. The latter finding, however, seems to be influenced by differences in pre-stimulus reference alpha power, since individuals scoring high on the subjective anger ratings exhibited significantly lower right- than left-hemispheric alpha power at baseline (interaction between HEMISPHERE and ANGER, *F* (1, 79) = 4.62, *p* < .05, *η*_*p*_^*2*^ = .06).

### Behavioral results

Descriptive statistics of the performance measures of the AUT (fluency and originality) and the RIT (fluency and flexibility) are presented in Table 1.

**Table 1:** Descriptive statistics of the performance measures of the Alternative Uses Test (AUT) and Reappraisal Inventiveness Test (RIT)

	Min	Max	*M*	*SD*
AUT fluency	1.75	12.50	5.53	2.15
AUT originality	2.04	3.27	2.87	0.22
RIT fluency	1.25	7.56	4.14	1.43
RIT flexibility	1.19	5.25	2.96	0.84

The fluency score of the AUT was significantly correlated with the RIT fluency score (*r* = .60, see Table 2), and both RIT and AUT fluency were significantly associated with the total score of the verbal imagination subscales of the BIS test (Jäger et al., 1997), a widely used and proven psychometric measure of verbal divergent thinking ability. No significant correlations were found with AUT originality.

**Table 2:** Intercorrelations between performance measures of the Alternative Uses Test (AUT) and Reappraisal Inventiveness Test (RIT), and correlations with the verbal imagination subscales of the Berlin Intelligence Structure (BIS) test

	AUT fluency	AUT originality	RIT fluency	RIT flexibility	BIS
AUT fluency		.00	.60**	.55**	.52**
AUT originality			.10	.13	.08
RIT fluency				.88**	.53**
RIT flexibility					.40**

** *p* < .01

## Discussion

Cognitive reappraisal was generally associated with a similar pattern of alpha oscillations as observed in conventional verbal creative ideation. This was particularly apparent in the finding that both the RIT and the AUT exhibited comparatively strong increases in alpha power at prefrontal sites. There were, however, also some important differences between cognitive reappraisal and conventional creative ideation, as the former yielded significantly larger alpha power increases at frontopolar sites, while performance of the AUT was associated with stronger alpha power increases at more posterior (i.e., central) cortical sites.

In functional imaging studies the prefrontal cortex has consistently been found to be implicated in various creativity-related task demands (Gonen-Yacoovi et al., 2013), hinting at the crucial role of executive processes in creative thought. Such processes may include effective working-memory processing, the inhibition of dominant associations or prepotent response tendencies, cognitive control (Beaty & Silvia 2012; Benedek et al., 2012, 2014; Gilhooly et al., 2007), or conceptual expansion, the widening of structures of existing concepts (Abraham et al., 2012). EEG studies nicely complement this picture. Alpha power increases at prefrontal sites, which have consistently been observed during verbal creative thinking demands (Fink & Benedek, 2014), are thought to indicate top-down control by actively inhibiting task-irrelevant activity such as irrelevant sensory processing or the retrieval of interfering information (e.g., Klimesch et al., 2007; Sauseng et al., 2005). Such processes may be generally relevant in tasks involving high internal processing demands – such as the generation of alternative uses or the generation of creative reappraisals to given anger-provoking situations.

In addition to the processes involved in conventional idea generation, cognitive reappraisal of emotion-eliciting situations or stressful events requires individuals to inhibit (disengage from) negative emotional aspects of a situation, and such a regulation of an ongoing emotional response might strongly draw on cognitive control processes (e.g., Joormann & Gotlib, 2010; Pe et al., 2013; Rowland et al., 2013; Weber et al., 2014). Additionally, cognitive reappraisal involves switching (shifting attention) towards emotionally neutral aspects, and updating the affective value and motivational relevance of the situation (cf. Malooly et al., 2013). Such higher order cognitive processes are known to be closely linked to functions of the prefrontal cortex, in part specifically with its most rostral parts (Bechara et al., 2000; Kalisch, 2009; Kringelbach & Rolls, 2004; Ochsner & Gross, 2005, 2008). Interestingly, the generation of reappraisals was associated with stronger alpha power increases at frontopolar sites than the generation of alternative uses, suggesting that the generation of reappraisals in anger-eliciting situations may more strongly rely on controlled information processing than the mere generation of alternative uses. In this particular context, one might assume that the more a situation becomes emotion-eliciting, the more the demands on cognitive control over emotional content increase. In fact, the more individuals experienced the presented RIT stimuli as being anger evoking, the stronger were the increases in alpha power at prefrontal sites (Fig. 5). Participants who experienced the presented RIT stimuli as being more anger evoking also generally exhibited stronger right- than left-hemispheric alpha power increases (cf. Balconi & Mazza, 2009). It should, however, be noted that participants with higher anger ratings already showed lower right- than left-hemispheric alpha power during the reference interval, which is nicely in line with consistent evidence from affective EEG laterality research focusing on states of anger (Harmon-Jones et al., 2010).

While the generation of cognitive reappraisals yielded stronger alpha power increases at prefrontal sites, the AUT was associated with stronger alpha power increases at more posterior (central) cortical sites (see Fig. 3). A diffuse and widespread pattern of alpha power increases at posterior cortical sites have quite consistently been observed in tasks involving creative thinking demands (Benedek et al., 2011, 2014; Fink, et al. 2009a, b, 2011; Schwab et al., 2014). In particular, alpha power increases over the right posterior cortex, as was tentatively also seen in this study, appear to be more specific to the process of creative ideation than alpha power increases at prefrontal sites (Benedek et al., 2011, 2014; Fink & Benedek, 2014). It has been suggested that such a finding could indicate an intense state of internal or task-focused attention, which may facilitate efficient search, retrieval, and integration of information from internal memory representations (Benedek et al., 2014; Fink & Benedek, 2014; see also Cabeza et al., 2008; Wagner et al., 2005). In light of recent functional imagining studies, which highlight the interplay of controlled and spontaneous imaginative modes of information processing during creative ideation (Beaty et al., 2015, 2016; Jung et al., 2013), it has been suggested that alpha power increases could also reflect default-mode or spontaneous imaginative thought, and such processes may be more attenuated the more controlled information processing is required (Mok, 2014). According to Beaty et al. (2015), spontaneous imaginative thought is closely linked to the brain’s default mode processes, including internally directed or self-generated thought such as mind-wandering, future thinking, perspective taking, and mental simulation (p.2). Thus, the finding of lower alpha power increases during cognitive reappraisal at more posterior cortical sites may also support the idea of a reduction of spontaneous imaginative towards more controlled modes of information processing during cognitive reappraisal as compared to idea generation without emotional component, characterized by an intense engagement in or the internal representation of an emotional event.

This is to our very best knowledge the first study that investigated functional patterns of EEG activity during the generation of reappraisals in relation to conventional idea generation without emotional component. The findings support the importance of cognitive control processes in the generation of reappraisals. However, several issues need to be more specifically addressed in future research. This study used a procedure in which only brain activity patterns immediately prior to idea generation (or prior to responding, respectively) were investigated. While the overall pattern of findings is quite similar to other EEG alpha studies using different experimental designs (e.g., Benedek et al., 2011, 2014; Fink, et al. 2009a, b, 2011), future studies should also investigate possible time-related changes of alpha power during conventional creative ideation and the generation of reappraisals. This is motivated by recent EEG studies (Jaarsveld et al., 2015; Schwab et al., 2014) revealing that alpha activity during creative idea generation seems to follow a characteristic time course, namely a general increase of alpha power at the beginning of idea generation, followed by a decrease and finally by a re-increase of alpha prior to responding. Such time-related effects in alpha power may possibly reflect different stages of the creative thinking process. Other designs applying fixed-idea generation periods (e.g., Fink, et al. 2009a; Schwab et al., 2014) are more powerful for studying the time course of brain activity patterns during creative cognition. Time-related effects might be also be expected concerning the originality of ideas. Benedek and Neubauer (2013) showed that in a word-association task both high- and low-creative individuals start with highly common responses and with increasing time they generate increasingly uncommon associations. In this study only an aggregated measure of AUT originality over the entire idea generation period was available, which may be a possible reason why in this study no effects related to originality were found, neither on the behavioral level nor at the level of the brain (note that in the majority of EEG and fMRI studies in our laboratory, task instructions stressed quality/originality to a stronger extent than in this study, see, e.g., Fink et al., 2015). Related to this, future studies should also consider scoring the AUT and the RIT on the same dimensions (including fluency, flexibility, and originality), in order to facilitate fine-grained comparisons of tasks at the behavioral/performance level. In addition, this study included only female participants, and future research in this field may be interested in also looking at potential sex differences with respect to the generation of reappraisals to emotional events, both psychometrically and at the level of the brain. Finally, future studies should also investigate creative ideation and cognitive reappraisal with other brain imaging methods such as fMRI, in order to learn more about the complex neural networks that are general to creative ideation and specific to idea generation in an affective context. In this particular context, studies would also benefit from the inclusion of another control task outside the realm of creativity and divergent thinking, in order to address the issue of the specificity versus generality of the cognitive and neural processes implicated in these tasks more precisely. For instance, such an approach would allow assessment of the extent to which the higher prefrontal alpha power increases in the RIT than in the AUT simply stem from higher demands on top-down inhibition or cognitive control processes implicated in this task, and/or from processes that are more specific to emotional information processing. Such an approach would additionally benefit from the conjoint use of EEG and fMRI methods, which should allow for a more precise assessment of the specific processes and mechanisms involved in the performance of these tasks, and, even more importantly, also facilitate the assessment of the manifold interactions with potential subcortical reappraisal systems (Ochsner & Gross, 2005).

Taken together, the findings of this study suggest that the generation of reappraisals and conventional creative ideation rely on quite similar functional patterns of brain activity, but also show some important differences. In line with relevant literature in this field, which highlights the crucial role of the prefrontal cortex in the emotion regulation domain (e.g., Ochsner & Gross, 2005, 2008), cognitive reappraisal yielded stronger alpha power increases at prefrontal sites than conventional creative idea generation without an emotional component, along with lower alpha power at more posterior (central) cortical sites, indicating more intense demands on controlled information processing when creative ideation operates in an affective context. Beyond extending the scope of research in the field of creativity, the present findings may help to stimulate the development of evidence-based strategies to enhance burdened individuals' capacities to use cognitive reappraisal, or to match patients to more appropriate treatments according to their individual deficits in relevant brain functions (e.g., Smoski et al., 2014).
